# Perceived Food Label Literacy and Use in Relation to Demographic Characteristics and Food Consumption Among Adults in Seoul: Analysis of Data from the Seoul Food Survey 2025

**DOI:** 10.3390/nu18182941

**Published:** 2026-09-08

**Authors:** Linxi Huang, Jihyun Yoon

**Affiliations:** 1 Department of Food and Nutrition, Seoul National University, Seoul 08826, Republic of Korea; 2 Research Institute of Human Ecology, Seoul National University, Seoul 08826, Republic of Korea; 3 Advanced Institute of Convergence Technology, Seoul National University, Suwon 16229, Republic of Korea

**Keywords:** nutrition labeling, food literacy, dietary behavior, urban health, Korea

## Abstract

**Background/Objectives:** This study aimed to identify demographic factors associated with perceived food label literacy and use among adults in Seoul and to examine differences in food consumption across perceived food label literacy and use groups. **Methods**: This study analyzed secondary data from the Seoul Food Survey 2025, which included 3024 adults aged ≥18 years. Perceived food label literacy and use were each dichotomized into high and low levels, yielding four groups based on their combination. Logistic regression and analysis of covariance were used to examine demographic factors and differences in food consumption. **Results**: The proportions of the high literacy–high use, high literacy–low use, low literacy–high use, and low literacy–low use groups were 37.5%, 21.4%, 12.1%, and 28.9%, respectively. Compared with the high literacy–high use group, men were more likely than women, and individuals with lower educational levels were more likely than those with a college graduation or higher to belong to both the high literacy–low use and low literacy–low use groups. The low literacy–low use group reported more frequent consumption of several processed foods, such as high-fat bread/snacks and sugar-sweetened beverages, and less frequent consumption of several fresh foods, such as fruits and whole grains, than at least one other group (*p* < 0.05). **Conclusions:** Men and individuals with lower educational levels emerged as potential priority populations for food label interventions addressing both perceived food label literacy and use. Those with low literacy and low use showed less favorable food consumption patterns.

## 1. Introduction

As processed and packaged foods account for an increasing proportion of daily food consumption worldwide [1,2], consumers increasingly use nutrition information to guide healthier food selection [3,4,5]. Food labels provide details about the nutrient content of foods and have become an important public health strategy to promote healthy eating behaviors [3,6]. Evidence indicates that food labeling improves consumer understanding of the nutritional quality of food [5]. Food label use has also been associated with healthier food choices such as higher consumption of vegetables and fruits and lower intake of unhealthy foods [7,8,9]. Furthermore, higher nutrition literacy is associated with greater food label use and healthier food-related decision-making [7,10]. 

The impact of food labels relies less on information availability than on consumer comprehension and application. However, previous studies have often treated food label literacy and food label use as closely related constructs, implicitly assuming that higher literacy translates into greater food label use [7,11]. Food label literacy and food label use are conceptually related but distinct constructs. While perceived food label literacy generally reflects the ability to understand nutrition information, use reflects whether individuals actively apply that information during food purchasing decisions. Nevertheless, emerging evidence suggests that understanding nutrition information does not necessarily translate into actual use during food purchasing or healthier food choices [12,13]. This discrepancy suggests that perceived food label literacy and use are related but distinct dimensions of nutrition behavior rather than interchangeable constructs [14]. Recognizing this distinction is important, as the two may exhibit different associations with food consumption patterns [7].

Despite growing interest in food labeling, most previous studies have examined nutrition-related literacy and food label use separately in relation to dietary behaviors, rather than jointly. For example, studies utilizing earlier waves of the Seoul Food Survey demonstrated that food literacy is associated with food intake among adults in Seoul [15,16], while analyses using the Korea National Health and Nutrition Examination Survey data have shown that food label reading is associated with nutrient intake among Korean adults [17]. Although nutrition-related literacy or knowledge may support food label use, evidence indicates that understanding and use are not necessarily consistently aligned [14,18]. However, examining these constructs separately may overlook distinct response categories. Individuals with high perceived food label literacy may not actively use labels during food purchasing, whereas others may frequently consult labels despite having limited understanding. Joint consideration of related literacy and use measures may help characterize heterogeneous response patterns. Accordingly, jointly considering perceived literacy and use may help identify concordant and discordant combinations that would not be apparent when the two constructs are examined separately.

To address this gap, this study aimed to identify demographic characteristics associated with different combinations of perceived food label literacy and use among adults in Seoul and to examine differences in food consumption across these groups. The findings may inform targeted food label interventions that address both perceived food label literacy and use to promote healthier food consumption patterns.

## 2. Subjects and Methods

### 2.1. Data Source and Subjects

This study analyzed secondary data from the Seoul Food Survey 2025, a representative population-based cross-sectional survey conducted by the Seoul Metropolitan Government from 15 September to 31 October 2025. The Seoul Food Survey is an official statistics survey approved under the Statistics Act of Korea (approval no. 201017) and has been conducted annually to assess food-related perceptions, dietary behaviors, and overall dietary life among Seoul residents.

The survey instrument consisted of six domains: food intake, dietary practices, food purchasing, food safety, health and happiness, and food policies and programs, as well as respondents’ demographic characteristics. The present study used variables measuring perceived food label literacy, food label use, food consumption frequency, and demographic characteristics.

The survey employed a stratified cluster sampling design to obtain a representative sample of adults in Seoul. Clusters were first selected using probability proportional systematic sampling, followed by systematic sampling of households within each cluster. Eligible respondents included adults aged 18 years or older living in the selected households. Data were collected through in-home, face-to-face interviews, with a self-administered questionnaire option available for participants who preferred not to complete a face-to-face interview. Among 3495 eligible respondents, 3024 completed the survey, corresponding to a completion rate of 86.5%. Reasons for non-completion included short-term travel or absence (8.1%), long-term travel or absence (2.7%), residence outside Seoul (0.8%), and other reasons (1.9%). The present secondary analysis included all 3024 respondents who completed the survey, with no additional exclusions. The original survey protocol was reviewed and approved by the Institutional Review Board of the Korean National Institute for Bioethics Policy (IRB approval number: P01-202509-01-042), and written informed consent was obtained from all participants prior to data collection.

### 2.2. Perceived Food Label Literacy and Use

Perceived food label literacy was measured using a single self-reported item, “I am able to understand food information (e.g., ingredient lists and nutrition labeling) on processed foods”, rated on a 5-point Likert scale ranging from 1 (strongly disagree) to 5 (strongly agree; mean score = 3.58). Responses were dichotomized based on explicit agreement. Respondents selecting “agree” or “strongly agree” (scores 4–5) were classified as having high perceived food label literacy, whereas those selecting “strongly disagree,” “disagree,” or “neither agree nor disagree” (scores 1–3) were classified as having low perceived food label literacy. This cutoff distinguished explicit agreement (scores 4–5) from neutrality or disagreement (scores 1–3) rather than defining objective literacy thresholds. Neutral responses were grouped into the latter category because it lacked explicit endorsement of perceived understanding.

Food label use was also assessed using a single self-reported item, “I check food information (e.g., ingredient lists and nutrition labeling) when purchasing processed foods” (mean score = 3.44), using the same response scale and classification criteria. Scores of 4–5 were classified as high use, and scores of 1–3 were classified as low use. This classification distinguished respondents who explicitly reported checking food information from those who did not explicitly report doing so when purchasing food. Although the survey item included nutrition labeling as an example, it referred broadly to food information, including ingredient lists. Accordingly, this construct is hereafter termed food label use and should be interpreted as self-reported use of on-package food information rather than exclusive use of nutrition labels.

Based on the levels of perceived food label literacy and use, respondents were subsequently categorized into four mutually exclusive groups: high literacy–high use, high literacy–low use, low literacy–high use, and low literacy–low use. These groups were used as analytical categories derived from dichotomized responses to the two single self-reported items and were not interpreted as behavioral phenotypes or consumer profiles.

### 2.3. Food Consumption

Food consumption was assessed using self-reported frequencies of consuming selected food items during the previous month. The selected food items were categorized into processed foods (processed meat, high-fat bread and snacks, fast food, sugar-sweetened beverages, zero-sugar beverages, foods with added alternative sweeteners, cola, energy drinks, and convenience store foods) and fresh foods (raw vegetables, fruits, milk and dairy products, fish, soybeans and tofu, nuts, and whole grains).

Because consumption frequencies were originally reported using different time units (per month, per week, or per day), all responses were standardized to weekly frequencies to ensure comparability across food items. Responses indicating almost no consumption were assigned a value of 0 times/week. For responses reported as ranges, the midpoint of each range was used (e.g., 1–3 times/week was converted to 2 times/week). Monthly frequencies were first represented by the midpoint of the corresponding response range and then divided by 4 to obtain weekly frequencies, whereas weekly frequencies were represented by the midpoint of the corresponding range. Daily frequencies were converted to weekly frequencies by multiplying the daily frequency by 7. These standardized frequencies represent consumption frequency only and do not reflect portion size, quantitative dietary intake, or overall dietary quality.

### 2.4. Demographic Characteristics

Demographic characteristics included sex, age, education level, household type, occupation, and monthly household income. Age was categorized into three groups: younger adults (18–34 years), middle-aged adults (35–64 years), and older adults (≥65 years). Education level was classified as middle school graduation or less, high school graduation, and college graduation or higher. Household type was categorized as living alone or living with others. Occupation was grouped into workers, students, and homemakers/others. Monthly household income was categorized as <3.5, 3.5 to <5.0, and ≥5.0 million KRW.

### 2.5. Statistical Analysis

All statistical analyses considered the complex sample design of the Seoul Food Survey 2025, including stratified clusters, primary sampling units, and sampling weights to obtain population-representative estimates. Descriptive statistics were presented as unweighted numbers and weighted percentages for categorical variables and weighted means with standard errors for continuous variables.

Differences in demographic characteristics across perceived food label literacy and use groups were examined using Rao–Scott χ^2^ tests. Differences in food consumption across these groups were evaluated by comparing mean weekly consumption frequencies for each food category using analysis of covariance based on general linear models, with adjustment for sex, age group, educational level, household type, occupation, and monthly household income. Overall group differences were adjusted for multiple testing using the Benjamini–Hochberg false discovery rate (FDR) procedure. For the four-group analysis, pairwise comparisons were performed using Bonferroni adjustment when the overall FDR-adjusted *p*-value was <0.05. Model assumptions were assessed using residual diagnostics, including Q–Q plots and residual-versus-fitted plots. Because some food consumption outcomes showed departures from normality, sensitivity analyses were conducted using log-transformed weekly consumption frequencies.

Separate binary logistic regression models were fitted to identify demographic factors associated with low perceived food label literacy and low food label use, respectively. A multinomial logistic regression modelwas performed to identify demographic factors associated with the four groups defined by perceived food label literacy and use: high literacy–high use, high literacy–low use, low literacy–high use, and low literacy–low use. The high literacy–high use group served as the reference category. All regression models included sex, age group, education level, household type, occupation, and monthly household income as independent variables. Results are presented as odds ratios (ORs) and 95% confidence intervals (CIs). Sensitivity analyses using an alternative cutoff (scores 1–2: low, scores 3–5: high) were conducted to assess the robustness of the findings regarding the dichotomization of perceived food label literacy and use.

All analyses were performed using SAS software, version 9.4 (SAS Institute Inc., Cary, NC, USA), specifically utilizing PROC SURVEYFREQ, PROC SURVEYMEANS, PROC SURVEYREG, and PROC SURVEYLOGISTIC. Statistical significance was determined using a two-sided *p*-value < 0.05.

## 3. Results

### 3.1. Demographic Characteristics of the Respondents

Table 1 and Table 2 present the demographic characteristics of the respondents. Overall, 59.0% of respondents were classified as having high perceived food label literacy, whereas 41.0% were classified as having low perceived food label literacy. For food label use, 49.6% were classified as high users, while 50.4% were classified as low users. When perceived food label literacy and use were considered jointly, 37.5% of respondents belonged to the high literacy–high use group, 21.4% to the high literacy–low use group, 12.1% to the low literacy–high use group, and 28.9% to the low literacy–low use group.

Demographic characteristics significantly differed according to perceived food label literacy and use levels. Lower perceived food label literacy and lower food label use were more prevalent among men, older adults, and individuals living alone. In addition, lower food label use was more prevalent among individuals with middle school graduation or less (all *p* < 0.05).

When perceived food label literacy and use were examined jointly, significant differences in sex, age group, education level, and household type were observed across the four groups (all *p* < 0.01). The low literacy–low use group had the highest proportion of men (59.7%), older adults (28.0%), individuals with a middle school graduation or less (15.5%), and those living alone (35.7%), whereas the high literacy–high use group had the highest proportion of women (62.3%), middle-aged adults (57.2%), individuals with a college graduation or higher (64.0%), and those living with others (76.3%). No significant differences were observed in occupation or monthly household income.

### 3.2. Factors Associated with Perceived Food Label Literacy and Use

Table 3 presents the results of separate logistic regression analyses examining demographic factors associated with perceived food label literacy and use. For perceived food label literacy, men were significantly more likely than women to report low perceived food label literacy (OR = 1.71, 95% CI: 1.30–2.26). Individuals living alone were also more likely than those living with others to report low perceived food label literacy (OR = 1.44, 95% CI: 1.01–2.05). For food label use, men were more likely than women (OR = 2.16, 95% CI: 1.64–2.86), older adults aged ≥65 years were more likely than younger adults aged 18–34 years (OR = 1.62, 95% CI: 1.03–2.57), individuals with a middle school graduation or less were more likely than those with a college graduation or higher (OR = 2.09, 95% CI: 1.26–3.47), and individuals living alone were more likely than those living with others (OR = 1.98, 95% CI: 1.46–2.70) to report low food label use. In contrast, adults with a monthly household income below 3.5 million KRW (OR = 0.53, 95% CI: 0.37–0.76) or between 3.5 and <5.0 million KRW (OR = 0.64, 95% CI: 0.47–0.86) were less likely to report low food label use than those with a monthly household income of ≥5.0 million KRW.

Sensitivity analyses using the alternative cutoff (scores 1–2: low; scores 3–5: high) showed directionally consistent associations for sex and education level, whereas associations with other demographic characteristics varied across operationalizations (Supplementary Table S1).

Table 4 presents the results of the multinomial logistic regression analysis of the four perceived food label literacy and use groups, with the high literacy–high use group as the reference category. Men were more likely than women to belong to the high literacy–low use group (OR = 1.81, 95% CI: 1.31–2.50) and the low literacy–low use group (OR = 2.73, 95% CI: 1.87–3.97). Adults with a middle school graduation or less were more likely than those with a college graduation or higher to belong to the high literacy–low use group (OR = 2.65, 95% CI: 1.32–5.35) and the low literacy–low use group (OR = 2.39, 95% CI: 1.23–4.65). Individuals living alone were more likely than those living with others to belong to the high literacy–low use group (OR = 1.60, 95% CI: 1.05–2.44) and the low literacy–low use group (OR = 2.25, 95% CI: 1.48–3.44). Older adults were more likely than younger adults to belong to the low literacy–low use group (OR = 1.97, 95% CI: 1.11–3.47). Workers were more likely than homemakers/others to belong to the low literacy–high use group (OR = 2.15, 95% CI: 1.22–3.77). In contrast, adults with a monthly household income below 3.5 million KRW were less likely than those with a monthly household income of ≥5.0 million KRW to belong to the low literacy–low use group (OR = 0.48, 95% CI: 0.29–0.82).

### 3.3. Food Consumption According to Perceived Food Label Literacy and Use

Table 5 presents weekly food consumption frequencies according to perceived food label literacy and use. Overall, after adjustment for demographic characteristics and multiple testing, respondents with low perceived food label literacy reported more frequent consumption of several processed foods and less frequent consumption of several fresh foods than those with high perceived food label literacy. Specifically, respondents with low perceived food label literacy consumed processed meat, high-fat bread and snacks, cola, and energy drinks more frequently and fruits, milk and dairy products, and whole grains less frequently than those with high perceived food label literacy (all FDR-adjusted *p* < 0.05). For food label use, respondents with low use consumed high-fat bread and snacks more frequently and nuts and whole grains less frequently than those with high use (all FDR-adjusted *p* < 0.05).

As shown in Table 6, when perceived food label literacy and use were examined jointly, significant overall group differences were observed for several processed and fresh foods after adjustment for demographic characteristics and multiple testing. Respondents with both low literacy and low use showed more frequent consumption of several processed foods, including high-fat bread and snacks, sugar-sweetened beverages, cola, and energy drinks, and less frequent consumption of raw vegetables, fruits, milk and dairy products, and whole grains than at least one of the other groups based on Bonferroni-adjusted pairwise comparisons. The food consumption patterns of the two discordant groups varied across food categories. Among the food categories examined, a pronounced between-group difference was observed for whole-grain consumption, ranging from 5.54 times per week in the high literacy–high use group to 3.89 times per week in the low literacy–low use group (FDR-adjusted *p* < 0.001). For several other food categories, however, the absolute between-group differences in weekly consumption frequency were small despite statistical significance and should therefore be interpreted cautiously in terms of practical magnitude.

Sensitivity analyses using log-transformed weekly food consumption frequencies generally yielded patterns consistent with the primary analyses, although the statistical significance of some individual food categories varied after transformation (Supplementary Table S2).

## 4. Discussion

This study identified demographic characteristics associated with perceived food label literacy and use among adults in Seoul and examined differences in food consumption across these groups. Three main findings emerged. First, while most adults reported high perceived food label literacy, fewer than half reported high food label use, and just over one-third had both high literacy and high use. Second, demographic characteristics differed across perceived food label literacy and use groups. Men, individuals with lower educational levels, and those living alone were more likely to belong to both the high literacy–low use and low literacy–low use groups, whereas older adults were more likely to belong to the low literacy–low use group. Workers were more likely to belong to the low literacy–high use group. Third, food consumption patterns differed among the four groups, with a comparatively less favorable pattern across several food categories observed in the low literacy–low use group.

Perceived food label literacy was assessed by self-report rather than objective testing. Potential discrepancies between perceived and objective literacy may reflect overconfidence [19], prior familiarity with food labels [20], educational background [10], or health consciousness [21] and could attenuate or exaggerate the observed associations with demographic characteristics and food consumption. As these factors were not directly assessed in the present study, they should be regarded as possible explanations rather than demonstrated mechanisms.

Against this measurement context, sex showed the most consistent associations with perceived food label literacy and use across all models. Compared with women, men were significantly more likely to report low perceived food label literacy and low food label use and to belong to both the high literacy–low use and low literacy–low use groups. Overall, men consistently reported lower label use, and high perceived literacy did not necessarily coincide with high reported use among men. These findings align with prior research showing that women are more likely than men to read and use food labels when purchasing food [22,23,24,25,26]. This sex difference has been linked to higher health consciousness among women [25,27,28], higher perceived relevance of dietary information [25,29], and women’s more frequent involvement in household food provisioning [30,31].

The higher odds of belonging to the high literacy–low use group among men further highlight the potential discordance between perceived understanding and reported use. These findings suggest that interventions focusing solely on perceived food label literacy may not fully address the lower food label use observed among men [32]. Future studies should examine whether motivational or contextual factors, such as the perceived relevance of food labels, time pressure during food purchasing, and social norms surrounding food choices, may help explain the lower reported food label use observed among men.

Age-related differences were observed primarily in reported food label use. Older adults (≥65 years) were more likely than younger adults to report low food label use (OR = 1.62, 95% CI: 1.03–2.56). In the four-group analysis, older adults were more likely than younger adults to belong to the low literacy–low use group (OR = 1.97, 95% CI: 1.11–3.47). These findings are broadly consistent with previous studies reporting age-related differences in food label understanding and use [33,34,35]. Possible explanations proposed in previous studies include greater reliance on fresh or traditional foods, which may reduce opportunities to encounter food labels [36], and age-related visual limitations, which may make small-print information on food packaging more difficult to use [37]. Because these factors were not assessed in the present study, they remain hypotheses requiring further investigation.

Lower educational levels and living alone were associated with lower food label use. Individuals with a middle school graduation or less were more likely than those with a college graduation or higher to report low food label use and to belong to both the high literacy–low use and low literacy–low use groups. The association with the high literacy–low use group suggests that high perceived food label literacy did not necessarily correspond to high food label use among individuals with lower educational levels. Previous studies have shown that food label comprehension is particularly challenging among individuals with limited nutrition literacy and numeracy skills [14,38]. One possible explanation for this apparent discrepancy is that the present study assessed perceived rather than objective food label literacy.

Similarly, individuals living alone were more likely than those living with others to report low food label use and to belong to both low-use groups (high literacy–low use and low literacy–low use). One hypothesized explanation is a lower perceived need to consult food information when purchasing or preparing food for oneself, which may be consistent with previous findings among single-person households [39]. However, perceived need for food information was not assessed in the present study, and this explanation therefore requires further investigation.

An unexpected finding was that workers were more likely than homemakers or others to belong to the low literacy–high use group (OR = 2.15, 95% CI: 1.22–3.77). A previous study has reported that employment status is positively associated with food label use [40], although this relationship may vary according to socioeconomic and occupational characteristics. The reasons for this association could not be examined with the available data.

Monthly household income showed an unexpected association with food label use. Compared with adults with monthly household incomes of ≥5.0 million KRW, those in the lower-income categories were less likely to report low food label use, and those with incomes <3.5 million KRW were also less likely to belong to the low literacy–low use group. This contrasts with previous studies reporting higher food label use among higher-income groups [11,41]. However, the association with household income was no longer statistically significant under the alternative dichotomization. This cutoff-sensitive finding should therefore be interpreted cautiously and not used to inform policy targeting or public health planning.

Perceived food label literacy and food label use did not always co-occur, and this discordance may be obscured when either dimension is examined separately. Using the four-group classification, reported food consumption differed across the analytical categories defined by perceived food label literacy and food label use, providing a descriptive framework for characterizing this heterogeneity. These results are consistent with previous studies suggesting that exposure to nutrition information does not necessarily lead to meaningful comprehension or behavioral change [38,42]. Among the four groups, the low literacy–low use group reported more frequent consumption of high-fat bread and snacks, sugar-sweetened beverages, cola, and energy drinks, together with less frequent consumption of raw vegetables, fruits, milk and dairy products, and whole grains. This finding is broadly consistent with previous studies reporting associations between food label use or understanding and healthier food consumption or dietary behaviors [38,43,44]. The present study adds to this literature by showing differences in reported consumption across several processed and fresh food categories when perceived food label literacy and food label use were considered jointly. However, given the cross-sectional design, these associations should not be interpreted as evidence that low perceived food label literacy or food label use causes changes in food consumption.

The two discordant groups did not show consistent intermediate values between the two concordant groups; rather, their consumption frequencies varied across food categories. For example, the high literacy–low use group showed relatively high fruit, milk and dairy product, and whole-grain consumption but lower nut consumption, whereas the low literacy–high use group showed relatively high consumption of raw vegetables and nuts. These results suggest that perceived food label literacy and use may reflect related but distinct constructs rather than interchangeable aspects of a single construct [14]. These findings also highlight the value of considering concordance and discordance between perceived understanding and reported use when examining differences in reported food consumption across the four analytical categories.

Differences in food consumption across perceived food label literacy and use groups also varied by food category. Although some between-group differences were statistically significant, the absolute differences in weekly consumption frequency were small for several food categories. These findings should therefore be interpreted as modest differences in reported consumption rather than as large differences in overall dietary patterns. Consumption frequencies of high-fat bread and snacks, sugar-sweetened beverages, cola, and energy drinks exhibited relatively larger differences across groups, whereas no significant differences were observed for several items, including fast food, zero-sugar beverages, fish, and soybeans and tofu. Previous studies have suggested that nutrition information on packaged foods may be more readily encountered at the point of purchase and incorporated into food-related decisions [8,9,45]. This may provide one possible context for the differences observed across selected packaged-food categories in the present study; however, attention to nutrition information and the reasons underlying individual food choices were not directly assessed. Previous studies also suggest that fast-food choices may reflect convenience, affordability, and habitual preferences [46], while zero-sugar beverage choices may be related to perceived healthfulness, weight-management motivations, or marketing influences [47,48]. These factors were not assessed in the present study and should therefore be considered possible contextual explanations. These findings suggest that future food labeling interventions may benefit from addressing both perceived food label literacy and use, with particular attention to individuals in the low literacy–low use group.

This study has several strengths. First, it analyzed representative survey data from adults living in Seoul using complex sampling weights. Second, by jointly evaluating perceived food label literacy and use, this study identified four analytical categories, including concordant and discordant response patterns, and enabled comparisons of demographic characteristics and reported food consumption across these categories.

However, several limitations should be considered. First, perceived food label literacy and use were each assessed using a single self-reported item rather than validated multi-item or objective measures and therefore could not capture the multidimensional nature of these constructs (e.g., objective comprehension, comparison skills, and interpretation of food label information) or fully reflect actual behavior. Potential discrepancies between perceived and actual understanding could have attenuated or, alternatively, exaggerated the magnitude or direction of the observed associations. Moreover, because the item assessing food label use referred broadly to food information, it may capture broader food-information checking behavior rather than the use of food labels exclusively. In addition, dichotomization of the 5-point responses may have resulted in information loss and sensitivity to the selected cutoff, although a sensitivity analysis using an alternative cutoff yielded directionally consistent associations for sex and education level. Second, food consumption was assessed based on consumption frequency rather than portion size or quantitative dietary intake and therefore should not be interpreted as a measure of overall dietary quality or nutritional exposure. Third, although the analyses adjusted for major demographic characteristics, residual confounding by unmeasured factors cannot be excluded. Finally, the cross-sectional design precludes determination of the temporal direction of the observed associations and does not permit causal inference.

## 5. Conclusions

Perceived food label literacy and use were not consistently aligned among adults in Seoul, with just over one-third reporting both high literacy and high use. Demographic characteristics were differentially associated with combinations of perceived food label literacy and use, with men and individuals with lower educational levels emerging as potential priority populations for food label interventions. Adults with both low perceived literacy and low use reported relatively less favorable food consumption frequencies for several food categories. These findings suggest that future food label interventions may benefit from addressing both perceived food label literacy and use. Such interventions could incorporate behaviorally tailored strategies that consider the characteristics of different demographic groups to promote healthier food consumption patterns.

## Figures and Tables

**Table 1:** Characteristics of respondents by levels of perceived food label literacy and use.

Characteristics	Total	High Literacy	Low Literacy	*p*-Value	High Use	Low Use	*p*-Value
	(*n* = 3024)	(*n* = 1782)	(*n* = 1242)		(*n* = 1515)	(*n* = 1509)	
Group distribution (weighted row %)	-	59.0	41.0		49.6	50.4	
Sex				<0.001			<0.001
Men	1406 (47.9)	729 (42.2)	677 (55.9)		581 (39.9)	825 (55.7)	
Women	1618 (52.1)	1053 (57.8)	565 (44.1)		934 (60.1)	684 (44.3)	
Age group				0.006			<0.001
Younger adults (18–34 years)	476 (27.2)	294 (26.4)	182 (28.3)		260 (29.4)	216 (25.0)	
Middle-aged adults (35–64 years)	1830 (52.5)	1121 (56.6)	709 (46.5)		957 (56.4)	873 (48.5)	
Older adults (≥65 years)	718 (20.4)	367 (17.0)	351 (25.1)		298 (14.2)	420 (26.4)	
Education level				0.147			<0.001
Middle school graduation or less	179 (11.3)	95 (9.4)	84 (13.9)		67 (7.1)	112 (15.4)	
High school graduation	1339 (30.6)	760 (30.8)	579 (30.2)		632 (30.1)	707 (31.1)	
College graduation or higher	1506 (58.1)	927 (59.8)	579 (55.8)		816 (62.8)	690 (53.6)	
Household type				0.021			0.002
Living alone	732 (29.2)	390 (26.3)	342 (33.4)		330 (24.7)	402 (33.7)	
Living with others	2292 (70.8)	1392 (73.7)	900 (66.6)		1185 (75.3)	1107 (66.3)	
Occupation				0.564			0.056
Worker	2302 (73.1)	1345 (71.6)	957 (75.1)		1138 (71.9)	1164 (74.2)	
Student	54 (5.5)	31 (5.5)	23 (5.4)		35 (7.5)	19 (3.5)	
Homemaker/other	668 (21.5)	406 (22.9)	262 (19.5)		342 (20.6)	326 (22.3)	
Monthly household income (10,000 KRW)				0.259			0.184
<350	1012 (34.6)	554 (33.1)	458 (36.7)		476 (32.1)	536 (37.1)	
350 to <500	662 (19.6)	397 (18.9)	265 (20.6)		351 (21.1)	311 (18.1)	
≥500	1350 (45.8)	831 (48.0)	519 (42.8)		688 (46.8)	662 (44.8)	

Values are presented as unweighted numbers (weighted column %). All analyses accounted for the complex sampling design of the Seoul Food Survey 2025. Variables were assessed on a 5-point Likert scale (1–3 = low, 4–5 = high). Perceived food label literacy was measured using the following item: “I am able to understand food information (e.g., ingredient lists and nutrition labeling) on processed foods”; food label use, reflecting self-reported use of on-package food information, was measured using the following item: “I check food information (e.g., ingredient lists and nutrition labeling) when purchasing processed foods.” *p*-values were calculated using the Rao–Scott χ^2^ test.

**Table 2:** Characteristics of respondents according to perceived food label literacy and use groups.

Characteristics	Total	High Literacy— High Use	High Literacy— Low Use	Low Literacy— High Use	Low Literacy— Low Use	*p*-Value
	(*n* = 3024)	(*n* = 1101)	(*n* = 681)	(*n* = 414)	(*n* = 828)	
Group distribution (weighted row %)	-	37.5	21.4	12.1	28.9	
Sex						<0.001
Men	1406 (47.9)	394 (37.7)	335 (50.3)	187 (46.8)	490 (59.7)	
Women	1618 (52.1)	707 (62.3)	346 (49.7)	227 (53.2)	338 (40.3)	
Age group						<0.001
Younger adults (18–34 years)	476 (27.2)	191 (30.0)	103 (20.2)	69 (27.6)	113 (28.6)	
Middle-aged adults (35–64 years)	1830 (52.5)	713 (57.2)	408 (55.4)	244 (54.1)	465 (43.4)	
Older adults (≥65 years)	718 (20.4)	197 (12.8)	170 (24.4)	101 (18.3)	250 (28.0)	
Education level						0.001
Middle school graduation or less	179 (11.3)	47 (6.1)	48 (15.2)	20 (10.2)	64 (15.5)	
High school graduation	1339 (30.6)	452 (29.9)	308 (32.4)	180 (30.7)	399 (30.1)	
College graduation or higher	1506 (58.1)	602 (64.0)	325 (52.3)	214 (59.1)	365 (54.5)	
Household type						0.006
Living alone	732 (29.2)	225 (23.7)	165 (31.0)	105 (28.0)	237 (35.7)	
Living with others	2292 (70.8)	876 (76.3)	516 (69.0)	309 (72.0)	591 (64.3)	
Occupation						0.116
Worker	2302 (73.1)	814 (70.1)	531 (74.2)	324 (77.3)	633 (74.2)	
Student	54 (5.5)	26 (7.6)	5 (1.9)	9 (7.1)	14 (4.7)	
Homemaker/other	668 (21.5)	261 (22.3)	145 (23.9)	81 (15.6)	181 (21.1)	
Monthly household income (10,000 KRW)						0.188
<350	1012 (34.6)	323 (30.8)	231 (37.2)	153 (36.0)	305 (37.0)	
350 to <500	662 (19.6)	258 (19.8)	139 (17.4)	93 (25.3)	172 (18.6)	
≥500	1350 (45.8)	520 (49.4)	311 (45.4)	168 (38.7)	351 (44.4)	

Values are presented as unweighted numbers (weighted column %). All analyses accounted for the complex sampling design of the Seoul Food Survey 2025. Variables were assessed on a 5-point Likert scale (1–3 = low, 4–5 = high). Perceived food label literacy was measured using the following item: “I am able to understand food information (e.g., ingredient lists and nutrition labeling) on processed foods”; food label use, reflecting self-reported use of on-package food information, was measured using the following item: “I check food information (e.g., ingredient lists and nutrition labeling) when purchasing processed foods.” *p*-values were calculated using the Rao–Scott χ^2^ test.

**Table 3:** Factors associated with low perceived food label literacy and low food label use.

Characteristics	Low Food Label Literacy	Low Food Label Use
	**OR (95% CI)**	**OR (95% CI)**
Sex		
Men	1.71 (1.30–2.26) ***	2.16 (1.64–2.86) ***
Women (Ref.)	1.00	1.00
Age group		
Younger adults (18–34 years) (Ref.)	1.00	1.00
Middle-aged adults (35–64 years)	0.77 (0.54–1.11)	0.87 (0.60–1.26)
Older adults (≥65 years)	1.50 (0.95–2.35)	1.62 (1.03–2.57) *
Education level		
Middle school graduation or less	1.42 (0.78–2.57)	2.09 (1.26–3.47) **
High school graduation	1.07 (0.82–1.40)	1.23 (0.94–1.60)
College graduation or higher (Ref.)	1.00	1.00
Household type		
Living alone	1.44 (1.01–2.05) *	1.98 (1.46–2.70) ***
Living with others (Ref.)	1.00	1.00
Occupation		
Worker	1.32 (0.94–1.86)	0.94 (0.69–1.30)
Student	1.21 (0.53–2.79)	0.46 (0.20–1.06)
Homemaker/other (Ref.)	1.00	1.00
Monthly household income (10,000 KRW)		
<350	0.77 (0.51–1.16)	0.53 (0.37–0.76) **
350 to <500	1.03 (0.74–1.45)	0.64 (0.47–0.86) **
≥500 (Ref.)	1.00	1.00
Model fit statistics	Max-rescaled R^2^ = 0.055 Wald F = 5.31 (*p* < 0.001)	Max-rescaled R^2^ = 0.102 Wald F = 9.33 (*p* < 0.001)

Abbreviations: OR, odds ratio; CI, confidence interval; Ref., reference. Values were estimated using binary logistic regression models. The outcomes were low perceived food label literacy and low food label use, respectively, with the corresponding high group as the reference category (*n* = 3024). * *p* < 0.05; ** *p* < 0.01; *** *p* < 0.001.

**Table 4:** Factors associated with perceived food label literacy and use groups.

Characteristics	High Literacy—Low Use	Low Literacy—High Use	Low Literacy—Low Use
	OR (95% CI)		
Sex			
Men	1.81 (1.31–2.50) ***	1.25 (0.84–1.86)	2.73 (1.87–3.97) ***
Women (Ref.)	1.00	1.00	1.00
Age group			
Younger adults (18–34 years) (Ref.)	1.00	1.00	1.00
Middle-aged adults (35–64 years)	1.22 (0.79–1.90)	1.16 (0.67–2.02)	0.71 (0.45–1.14)
Older adults (≥65 years)	1.74 (0.94–3.22)	1.64 (0.82–3.26)	1.97 (1.11–3.47) *
Education level			
Middle school graduation or less	2.65 (1.32–5.35) **	1.83 (0.77–4.33)	2.39 (1.23–4.65) *
High school graduation	1.27 (0.90–1.79)	1.04 (0.69–1.59)	1.22 (0.86–1.72)
College graduation or higher (Ref.)	1.00	1.00	1.00
Household type			
Living alone	1.60 (1.05–2.44) *	0.96 (0.58–1.58)	2.25 (1.48–3.44) ***
Living with others (Ref.)	1.00	1.00	1.00
Occupation			
Worker	1.13 (0.74–1.73)	2.15 (1.22–3.77) **	1.15 (0.77–1.72)
Student	0.35 (0.10–1.26)	2.20 (0.69–6.99)	0.68 (0.24–1.90)
Homemaker/other (Ref.)	1.00	1.00	1.00
Monthly household income (10,000 KRW)			
<350	0.74 (0.49–1.11)	1.43 (0.84–2.44)	0.48 (0.29–0.82) **
350 to <500	0.77 (0.52–1.13)	1.69 (0.99–2.89)	0.70 (0.47–1.02)
≥500 (Ref.)	1.00	1.00	1.00
Model fit statistics	Max-rescaled R^2^ = 0.114, Wald F = 4.24 (*p* < 0.001)		

Abbreviations: OR, odds ratio; CI, confidence interval; Ref., reference. Values were estimated using a multinomial logistic regression model, with the high literacy–high use group serving as the reference category (*n* = 3024). * *p* < 0.05; ** p < 0.01; *** *p* < 0.001.

**Table 5:** Weekly food consumption frequencies by levels of perceived food label literacy and use.

Food Category	Total (*n* = 3024)	High Literacy (*n* = 1782)	Low Literacy (*n* = 1242)	*p*- Value	FDR-Adjusted *p*-Value	High Use (*n* = 1515)	Low Use (*n* = 1509)	*p*-Value	FDR-Adjusted *p*-Value
Processed foods									
Processed meat	1.37 (0.05)	1.14 (0.05)	1.41 (0.06)	0.002	0.007	1.20 (0.06)	1.31 (0.06)	0.189	0.253
High-fat bread/snacks	1.38 (0.05)	1.16 (0.04)	1.39 (0.07)	0.007	0.019	1.09 (0.05)	1.43 (0.05)	<0.001	<0.001
Fast food	1.10 (0.04)	0.97 (0.04)	1.09 (0.05)	0.062	0.108	0.95 (0.05)	1.08 (0.04)	0.065	0.113
Sugar-sweetened beverages	1.68 (0.08)	1.56 (0.08)	1.82 (0.13)	0.106	0.155	1.51 (0.08)	1.82 (0.11)	0.024	0.060
Zero-sugar beverages	0.70 (0.04)	0.66 (0.04)	0.59 (0.05)	0.392	0.448	0.59 (0.04)	0.68 (0.05)	0.259	0.319
Foods with added alternative sweeteners	0.48 (0.02)	0.43 (0.03)	0.50 (0.03)	0.137	0.182	0.42 (0.03)	0.49 (0.03)	0.091	0.132
Cola	0.98 (0.04)	0.77 (0.03)	1.06 (0.06)	<0.001	0.001	0.81 (0.04)	0.97 (0.05)	0.024	0.060
Energy drinks	0.64 (0.04)	0.52 (0.04)	0.75 (0.05)	0.001	0.005	0.53 (0.05)	0.70 (0.05)	0.013	0.051
Convenience store foods	0.65 (0.03)	0.55 (0.04)	0.68 (0.05)	0.062	0.108	0.61 (0.05)	0.59 (0.04)	0.774	0.774
Fresh foods									
Raw vegetables	4.29 (0.08)	4.57 (0.11)	4.27 (0.11)	0.068	0.108	4.63 (0.11)	4.28 (0.11)	0.026	0.060
Fruits	4.40 (0.09)	4.75 (0.10)	4.28 (0.12)	0.003	0.011	4.69 (0.10)	4.43 (0.10)	0.071	0.113
Milk and dairy products	4.48 (0.09)	4.54 (0.12)	4.11 (0.12)	0.012	0.028	4.43 (0.14)	4.30 (0.11)	0.493	0.564
Fish	1.64 (0.04)	1.73 (0.06)	1.70 (0.06)	0.656	0.656	1.80 (0.06)	1.64 (0.05)	0.039	0.078
Soybeans and tofu	4.52 (0.07)	4.54 (0.08)	4.66 (0.10)	0.361	0.444	4.61 (0.09)	4.56 (0.08)	0.699	0.745
Nuts	1.88 (0.07)	1.96 (0.08)	2.03 (0.11)	0.587	0.626	2.19 (0.10)	1.79 (0.08)	0.001	0.007
Whole grains	4.62 (0.15)	5.38 (0.17)	4.06 (0.17)	<0.001	<0.001	5.26 (0.17)	4.44 (0.16)	<0.001	0.001

Values are presented as weighted means (SEs) for the total sample and adjusted means (SEs) for the groups. All weighted models accounted for the complex sampling design of the Seoul Food Survey 2025. *p*-values for group differences were obtained using general linear models adjusted for sex, age group, educational level, household type, occupation, and monthly household income and were adjusted for multiple testing across food groups using the Benjamini–Hochberg false discovery rate (FDR) procedure. Food consumption frequencies were standardized as times per week.

**Table 6:** Weekly food consumption frequencies according to perceived food label literacy and use groups.

Food Category	High Literacy– High Use (*n* = 1101)	High Literacy–Low Use (*n* = 681)	Low Literacy– High Use (*n* = 414)	Low Literacy–Low Use (*n* = 828)	*p*-Value	FDR-Adjusted *p*-Value
Processed foods						
Processed meat	1.12 (0.07)	1.18 (0.07)	1.41 (0.10)	1.42 (0.09)	0.012	0.028
High-fat bread/snacks	1.06 ^b^ (0.06)	1.33 ^a^ (0.07)	1.16 ^ab^ (0.10)	1.50 ^a^ (0.09)	<0.001	0.003
Fast food	0.92 (0.06)	1.05 (0.06)	1.06 (0.07)	1.11 (0.07)	0.139	0.185
Sugar-sweetened beverages	1.56 ^ab^ (0.10)	1.56 ^ab^ (0.12)	1.36 ^b^ (0.13)	2.02 ^a^ (0.17)	0.025	0.040
Zero-sugar beverages	0.60 (0.05)	0.75 (0.07)	0.55 (0.07)	0.62 (0.07)	0.238	0.272
Foods with added alternative sweeteners	0.40 (0.04)	0.46 (0.04)	0.46 (0.06)	0.51 (0.04)	0.320	0.342
Cola	0.78 ^b^ (0.04)	0.76 ^b^ (0.05)	0.91 ^ab^ (0.08)	1.13 ^a^ (0.08)	0.001	0.007
Energy drinks	0.50 ^b^ (0.06)	0.55 ^b^ (0.05)	0.61 ^ab^ (0.06)	0.82 ^a^ (0.08)	0.010	0.026
Convenience store foods	0.55 (0.05)	0.55 (0.04)	0.78 (0.08)	0.63 (0.07)	0.044	0.063
Fresh foods						
Raw vegetables	4.57 ^ab^ (0.13)	4.57 ^ab^ (0.17)	4.78 ^a^ (0.15)	4.04 ^b^ (0.15)	0.007	0.024
Fruits	4.72 ^a^ (0.12)	4.79 ^a^ (0.15)	4.58 ^ab^ (0.16)	4.15 ^b^ (0.15)	0.018	0.035
Milk and dairy products	4.45 ^ab^ (0.17)	4.70 ^a^ (0.18)	4.38 ^ab^ (0.22)	3.99 ^b^ (0.14)	0.020	0.036
Fish	1.80 (0.07)	1.62 (0.08)	1.79 (0.10)	1.65 (0.07)	0.226	0.272
Soybeans and tofu	4.57 (0.11)	4.47 (0.11)	4.71 (0.15)	4.63 (0.12)	0.584	0.584
Nuts	2.10 ^ab^ (0.11)	1.72 ^b^ (0.11)	2.45 ^a^ (0.23)	1.84 ^ab^ (0.12)	0.004	0.016
Whole grains	5.54 ^a^ (0.20)	5.13 ^ab^ (0.26)	4.41 ^bc^ (0.28)	3.89 ^c^ (0.21)	<0.001	<0.001

Values are presented as weighted means (SEs) for the total sample and adjusted means (SEs) for the four groups. All weighted models accounted for the complex sampling design of the Seoul Food Survey 2025. *p*-values for overall group differences were obtained using general linear models adjusted for sex, age group, educational level, household type, occupation, and monthly household income and were adjusted for multiple testing across food groups using the Benjamini–Hochberg false discovery rate (FDR) procedure. Pairwise comparisons between groups were performed using Bonferroni adjustment when the overall FDR-adjusted *p*-value was <0.05. Values within a row with different superscript letters (a, b, c) are significantly different (*p* < 0.05) based on Bonferroni-adjusted post hoc pairwise comparisons; values sharing a common superscript letter do not differ significantly. Food consumption frequencies were standardized as times per week.

## Data Availability

Publicly available datasets were analyzed in this study. The data analyzed in this study are publicly available from the Seoul Open Data Plaza at the following URL: https://data.seoul.go.kr/dataList/OA-22263/F/1/datasetView.do (accessed on 1 May 2026).

## Supplementary Materials

The following supporting information can be downloaded at https://www.mdpi.com/article/10.3390/nu18182941/s1, Table S1: Sensitivity analyses of demographic characteristics associated with perceived food label literacy and use using an alternative operationalization (scores 1–2: low; scores 3–5: high); Table S2: Sensitivity analyses of food consumption using log-transformed weekly consumption frequencies.

## Abbreviations

The following abbreviations are used in this manuscript:

CI	Confidence interval
FDR	False discovery rate
IRB	Institutional Review Board
KRW	Korean Won
OR	Odds ratio
Ref.	Reference
SE	Standard error
