# Supplementary material for: Perceived Food Label Literacy and Use in Relation to Demographic Characteristics and Food Consumption Among Adults in Seoul: Analysis of Data from the Seoul Food Survey 2025
Source: Nutrients. 2026 Sep 8;18(18):2941. doi: 10.3390/nu18182941 (PMC13610965; doi:10.3390/nu18182941)
Supplement: Supplementary file 1 [file nutrients-18-02941-s001.zip › nutrients-4505495-supplementary.pdf]

Supplementary Table S1. Sensitivity analyses of demographic characteristics associated with perceived food label literacy and use using an alternative operationalization (scores 1–2: low; scores 3–5: high).

Characteristics	Low food label literacy		Low food label use	
	**OR (95% CI)**	***p*-value**	**OR (95% CI)**	***p*-value**
Sex				
Men	**2.02 (1.25–3.24)**	0.004	**3.04 (2.02–4.58)**	<0.001
Women	Ref.		Ref.	
Age group				
Younger adults (18–34 years)	Ref.		Ref.	
Middle-aged adults (35–64 years)	0.87 (0.43–1.78)	0.698	1.06 (0.52–2.14)	0.873
Older adults (≥ 65 years)	1.35 (0.64–2.85)	0.426	1.65 (0.87–3.15)	0.126
Education level				
Middle school graduation or less	**3.63 (1.44–9.10)**	0.006	**2.96 (1.37–6.38)**	0.006
High school graduation	1.30 (0.77–2.20)	0.322	1.26 (0.86–1.84)	0.232
College graduation or higher	Ref.		Ref.	
Household type				
Living alone	1.41 (0.77–2.56)	0.263	1.71 (0.99–2.95)	0.056
Living with others	Ref.		Ref.	
Occupation				
Worker	**0.53 (0.33–0.88)**	0.013	0.80 (0.52–1.23)	0.312
Student	0.48 (0.09–2.63)	0.400	0.89 (0.21–3.83)	0.877
Homemaker/other	Ref.		Ref.	
Monthly household income (10,000 KRW)				
< 350	0.55 (0.17–1.74)	0.308	0.51 (0.22–1.21)	0.126
350 to < 500	0.72 (0.34–1.51)	0.381	0.64 (0.34–1.22)	0.177
≥ 500	Ref.		Ref.	
Model fit statistics	Max-rescaled R^2 = 0.088<br>Wald F = 4.28 ($p < 0.001$)		Max-rescaled R^2 = 0.090<br>Wald F = 8.32 ($p < 0.001$)	

Abbreviations: OR, odds ratio; CI, confidence interval; Ref., reference. Values were estimated using binary logistic regression models. The original 5-point score distributions [unweighted n (weighted %)] were as follows: perceived food label literacy, 1 = 3 (0.1%), 2 = 189 (7.0%), 3 = 1,050 (33.9%), 4 = 1,637 (53.1%), and 5 = 145 (5.9%); food label use (self-reported use of on-package food information, including ingredient lists and nutrition labeling), 1 = 17 (0.5%), 2 = 332 (11.7%), 3 = 1,160 (38.2%), 4 = 1,308 (42.4%), and 5 = 207 (7.2%). In the alternative dichotomization, scores of 1–2 were classified as low and scores of 3–5 as high. The resulting distributions [unweighted n (weighted %)] were 192 (7.1%) for low and 2,832 (92.9%) for high perceived food label literacy, and 349 (12.2%) for low and 2,675 (87.8%) for high food label use. The corresponding high group was used as the reference category for each outcome.

Supplementary Table S2. Sensitivity analyses of food consumption using log-transformed weekly consumption frequencies

Food category	Perceived food label literacy		Food label use		Perceived food label literacy and use groups	
	Original FDR-adjusted *p*	Log-transformed FDR-adjusted *p*	Original FDR-adjusted *p*	Log-transformed FDR-adjusted *p*	Original FDR-adjusted *p*	Log-transformed FDR-adjusted *p*
Processed foods						
Processed meat	0.007	0.004	0.253	0.272	0.028	0.016
High-fat bread/snacks	0.019	0.050	<0.001	0.001	0.003	0.006
Fast food	0.108	0.079	0.113	0.087	0.185	0.093
Sugar-sweetened beverages	0.155	0.198	0.060	0.087	0.040	0.102
Zero-sugar beverages	0.448	0.202	0.319	0.520	0.272	0.179
Foods with added alternative sweeteners	0.182	0.270	0.132	0.104	0.342	0.309
Cola	0.001	0.009	0.060	0.218	0.007	0.037
Energy drinks	0.005	0.003	0.051	0.050	0.026	0.016
Convenience store foods	0.108	0.079	0.774	0.636	0.063	0.037
Fresh foods						
Raw vegetables	0.108	0.222	0.060	0.071	0.024	0.016
Fruits	0.011	0.009	0.113	0.087	0.035	0.018
Milk and dairy products	0.028	0.037	0.564	0.470	0.036	0.038
Fish	0.656	0.779	0.078	0.071	0.272	0.134
Soybeans and tofu	0.444	0.202	0.745	0.842	0.584	0.498
Nuts	0.626	0.404	0.007	0.071	0.016	0.060
Whole grains	<0.001	<0.001	0.001	0.004	<0.001	<0.001

Weekly food consumption frequencies were log-transformed using log (x + 1). *p*-values for overall group differences were obtained using general linear models with the same survey design and covariate adjustment as in the primary analyses and were adjusted for multiple testing across food categories using the Benjamini–Hochberg false discovery rate (FDR) procedure. "Original" refers to analyses using untransformed weekly consumption frequencies, whereas "log-transformed" refers to sensitivity analyses using log (x + 1)-transformed weekly consumption frequencies.
